# Exercise-induced bronchoconstriction, temperature regulation and the role of heat shock proteins in non-asthmatic recreational marathon and half-marathon runners

**DOI:** 10.1038/s41598-019-39983-9

**Published:** 2019-03-12

**Authors:** Christine Bekos, Matthias Zimmermann, Lukas Unger, Stefan Janik, Andreas Mitterbauer, Michael Koller, Robert Fritz, Christian Gäbler, Jessica Didcock, Jonathan Kliman, Walter Klepetko, Hendrik Jan Ankersmit, Bernhard Moser

**Affiliations:** 1 Christian Doppler Laboratory for Cardiac and Thoracic Diagnosis and Regeneration, Vienna, Austria; 2 Medical University of Vienna, Comprehensive Cancer Center, Department of Obstetrics and Gynecology, Division of General Gynecology and Gynecologic Oncology, Vienna, Austria; 3 Sportordination, Alserstraße 28, Vienna, Austria; 4 Department of Surgery, Division of Thoracic Surgery, Medical University Vienna, Vienna, Austria

**Keywords:** Prognostic markers, Prognostic markers, Inflammation

## Abstract

Exercise is the most common trigger of bronchospasm. Heat shock protein (HSP) expression was linked to asthmatic patients. The prevalence and pathophysiology of exercise-induced bronchoconstriction (EIB) in non-professional non-asthmatic runners is unknown. We sought to investigate the frequency of EIB and cytokine changes in non-professional non-asthmatic marathon and half marathoners with and without EIB. Testing was performed before the marathon (baseline), immediately post-marathon at the finish area (peak), and 2–7 days after the marathon (recovery): immunosorbent assays for measurement of HSP70, blood count analysis, spirometry and temperature measurements. We experienced a decline in FEV1 of ≥10% in 35.29% of marathon and 22.22% of half marathon runners. Runners with EIB had significantly higher HSP70 serum concentrations at baseline than those without EIB (987.4 ± 1486.7 vs. 655.6 ± 1073.9; p = 0.014). Marathoners with EIB had significantly increased WBC before participating in the competition (7.4 ± 1.7 vs. 6.0 ± 1.5; p = 0.021). After recovery we found increased HSP70 serum concentrations in marathoners with EIB compared to those without (2539.2 ± 1692.5 vs. 1237.2 ± 835.2; p = 0.032), WBC (7.6 ± 1.8 vs. 6.4 ± 1.6; p = 0.048) and PLT (273.0 ± 43.0 vs 237.2 ± 48.3; p = 0.040). At all measured skin sites skin temperatures in runners were significantly lower immediately after participating in the competition when compared to temperature before the race (skin temperature baseline vs. peak: abdominal: 33.1 ± 0.2 vs. 30.0 ± 0.4; p < 0.001; upper arm: 31.6 ± 0.2 vs. 29.4 ± 0.3; p < 0.001; upper leg: 30.7 ± 0.3 vs. 29.4 ± 0.2; p = 0.014; lower leg: 30.6 ± 1.0 vs. 30.2 ± 1.5; p = 0.007). We found a higher than expected number of non-professional athletes with EIB. HSP70 serum concentrations and elevated WBC could indicate a predisposition to EIB.

## Introduction

Marathon and half-marathon running has become more and more popular during the last few decades. Since 1970 number of finishers at the New York city marathon are increasing steadily, starting from 55 in 1970 increasing to 50773 in 2017^1^. On the one hand regular moderate endurance training is one of the most striking strategies in prevention of obesity^2^, diabetes^3^, osteoporosis^4^, cancer^5^ and cardiovascular diseases^3^. On the other hand vigorous exhaustion such as marathon running leads to an increase of various proinflammatory markers and acute inflammatory response^6^.

Exercise-induced bronchoconstriction (EIB) describes the acute onset of bronchoconstriction occurring during or immediately after exercise. The prevalence varies from 5% to 20% in the general population^7–9^ but reaches almost 90% in patients with symptomatic asthma^10^. If exercise is the only identifiable trigger for bronchoconstriction, it is called EIB, with an estimated prevalence of 8–20% in the non-asthmatic population. The main mechanism leading to EIB is probably due to increased ventilation leading to airway dehydration and epithelial injury. Further this results in augmented osmolarity of the airway-lining fluid and hyperemia of the bronchial vasculature. Stimulated airway inflammatory cells release histamine, cysteine leukotrienes and prostaglandis resulting in airway smooth muscle contraction and airway edema^11,12^.

EIB is probably a result of changes in airway physiology initiated by a large volume of cool and dry air inhaled during vigorous activity^13,14^. Cooling the skin induces airway constriction via a somatic afferent-vagal efferent reflex arc^15^, while breathing cold air directly leads to bronchial narrowing by its actions on intra-airway thermodynamics^16^. When improving cardiovascular fitness, the minute ventilation required for a given level of exercise is reduced and the stimulus for bronchoconstriction is decreased. Moreover, bronchoconstriction is less likely in warmer and more humid inspired gas^17^. Patients are instructed to breathe through a loosely fitting scarf or mask when exercising in cold, dry condition^17^. Another explanation fort he pathogenesis of EIB relates to the epithelial injury resulting from inhaling poorly conditioned air at high flows for a long time or high volumes of irritant particles or gases^12^. In a trial investigating lung function during skin cooling in 10 individuals, chilling the skin, while core temperature was stable, produced mild airway obstruction^18^. Therefore we hypothesized that EIB might be much more frequent in patients with decreased skin temperature than in those participants with normal temperature.

Heat shock proteins (HSPs) are classified by their molecular weight into HSP27, 60, 70 and 90. HSPs can undertake a cell-protective role that may be induced by reactive oxygen species, cytokines, and hyperthermia. HSPs function as molecular chaperons to ensure correct folding, function and location. HSPs are inducible in various tissues and cell lines to maintain homeostasis, enable repair from injury and prevent cells from further damage^19,20^. The pathophysiological role of HSPs is already well known for malignancies^21^, autoimmune diseases^22^, and cardiovascular diseases^23^. HSP70 mRNA expression was detected in PBMCs of patients with asthma during acute episodes as well as in symptom-free patients^24^. Further it has been shown that in patients with asthma HSP 70 expression in airway epithelial, bronchial mucous cells and macrophages in bronchoalveolar lavage fluid was much higher compared to patients with chronic bronchitis and controls^25^.

The aim of this study was to investigate the incidence of EIB in non-asthmatic non-professional runners and to study the association of EIB and changes in cytokine concentrations, skin or core temperature.

## Results

### Demographic data

Thirty-four marathoners, 36 half-marathoners and 30 sedentary volunteers were included in this study. For further demographic details, running and training history please study the related publication^26^.

### Core and skin temperature measurements

There were neither significant differences in baseline core temperatures between M and HM runners nor at any of the other investigated time points (baseline, peak, recovery).

At all measured skin sites (abdomen, upper arm, upper and lower leg) skin temperatures in M and HM runners were significantly lower immediately after participating in the race compared to temperatures before the competition (skin temperature abdominal: M_baseline 33.14 ± 0.25 vs. M_peak 30.09 ± 0.41, p < 0.001; HM_baseline 33.15 ± 0.25 vs. HM_peak 30.44 ± 0.34, p < 0.001; skin temperature upper arm: M_baseline 31.68 ± 0.21 vs. M_peak 29.41 ± 0.39, p < 0.001, HM_baseline 31.73 ± 0.18 vs. HM_peak 28.79 ± 0.30, p < 0.001; skin temperature upper leg: M_baseline 30.70 ± 0.30 vs. M_peak 29.43 ± 0.27, p = 0.014, HM_baseline 30.01 ± 0.21 vs. HM_peak 28.51 ± 0.32, p = 0.001; skin temperature lower leg: HM_baseline 29.96 ± 0.17 vs. HM_peak 28.82 ± 0.31, p = 0.007).

An exception to this is the skin temperature measured at the lower leg in marathoners that did not change significantly after participating in the race.

The skin temperatures measured at the upper and lower leg were significantly lower in M and HM runners at rest when compared to sedentary controls (skin temperature upper leg: M_baseline 30.70 ± 0.30 vs. controls 32.06 ± 0.23, p = 0.007; HM_baseline 30.01 ± 0.21 vs. controls, p < 0.001; skin temperature lower leg: M_baseline 30.69 ± 0.18 vs. control 31.88 ± 0.25, p = 0.008; HM_baseline 29.96 ± 0.17 vs. control, p < 0.001) (Table 1).

**Table 1:** Core and skin temperature in 34 marathoners, 36 half-marathoners and 30 sedentary controls before the run (baseline), immediately after the run (peak) and after 2 to 7 days of recovery (recovery).

	M baseline	M peak	HM baseline	HM peak	Sedentary subjects	p-value
Core temperature	36.82 ± 0.81	36.30 ± 0.61	36.74 ± 0.39	36.07 ± 1.22	36.26 ± 1.89	0.020^a^
Skin temperature abdominal	33.14 ± 1.46	30.09 ± 2.34	33.15 ± 1.52	30.44 ± 2.03	32.50 ± 1.15	<0.001^a^
Skin temperature upper arm	31.68 ± 1.18	29.41 ± 2.18	31.73 ± 1.09	28.79 ± 1.76	32.26 ± 1.12	<0.001^a^
Skin temperature upper leg	30.70 ± 1.74	29.43 ± 1.55	30.01 ± 1.25	28.51 ± 1.91	32.06 ± 1.27	<0.001^a^
Skin temperature lower leg	30.69 ± 1.03	30.2 ± 1.51	29.96 ± 1.03	28.82 ± 1.81	31.88 ± 1.38	<0.001^a^

All results are reported as mean ± standard deviation. M, marathon; HM, half-marathon; baseline, 1–2 days before the run; peak, immediately after the run in the finishing area; recovery, after 2–7 days of recovery.

^a^One-way ANOVA.

Notably, temperature measurements were taken in a heated room at baseline (20 °C), in a tent at the finish line (9–11 °C outside)^27^ and at a heated room (22 °C) at the general hospital of Vienna at recovery.

### Spirometry

#### Forced expiratory volume after one second (FEV1)

**Figure 1:**
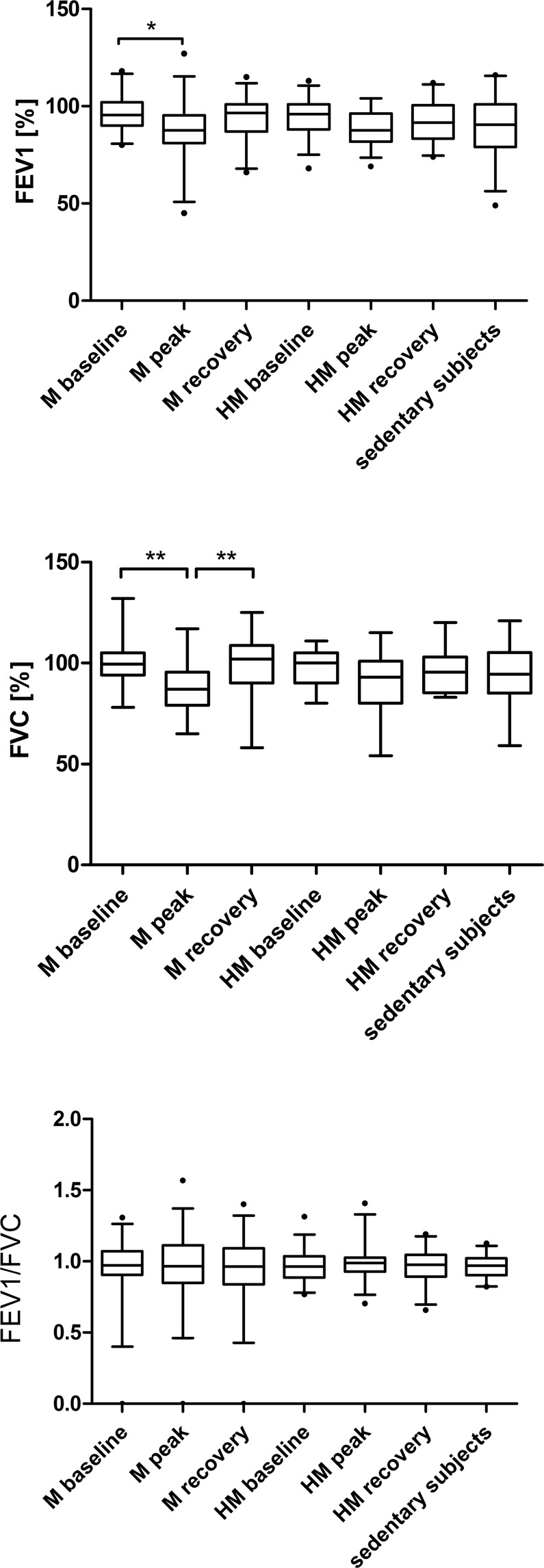
(**a**) Predicted FEV1% in marathoners and half-marathoners at baseline, peak and recovery compared to sedentary controls. Predicted FEV1% significantly decreased after participating in a M while remaining stable in HM. There were no differences detectable between M, HM and sedentary controls. M, marathon; HM, half-marathon; baseline, 1–2 days before the run; peak, immediately after the run in the finishing area; recovery, after 2–7 days of recovery; FEV1, forced expiratory volume after one second. *p < 0.05. (**b**) Predicted FVC % in marathoners and half-marathoners at baseline, peak and recovery compared to sedentary controls. Predicted FVC % significantly decreased after participating in a M and remained stable in HM. There were no differences detectable between M and HM runners and sedentary controls. M, marathon; HM, half-marathon; baseline, 1–2 days before the run; peak, immediately after the run in the finishing area; recovery, after 2–7 days of recovery; FVC, forced vital capacity. **p < 0.01. (**c**) FEV1/FVC ratio in marathoners and half-marathoners at baseline, peak and recovery compared to sedentary controls. FEV1/FVC ratio remained stable in M and HM runners. There were no differences detectable between M and HM runners and sedentary controls. M, marathon; HM, half-marathon; baseline, 1–2 days before the run; peak, immediately after the run in the finishing area; recovery, after 2–7 days of recovery; FVC, Forced vital capacity. **p < 0.01.

FEV1 decreased significantly immediately after M ([%predicted] M_baseline 96.00 ± 1.69 vs. M_peak 86.56 ± 2.84, p = 0.038) (Table 2). Significant intergroup differences are shown in Fig. 1a.

**Table 2:** Lung function testing in 34 marathoners, 36 half-marathoners and 30 sedentary controls before the run (baseline), immediately after (peak) and after 2 to 7 days of recovery.

	M baseline	M peak	M recovery	HM baseline	HM peak	HM recovery	Sedentary subjects	p-value
FVC	100.06 ± 11.27	87.61 ± 13.05	99.19 ± 14.12	98.09 ± 8.57	90.49 ± 12.94	96.16 ± 10.33	94.23 ± 15.18	<0.001^a^
FEV1	96.00 ± 9.53	86.56 ± 16.04	93.91 ± 11.38	94.49 ± 9.93	88.53 ± 9.11	91.81 ± 10.53	90.46 ± 16.10	0.018^a^
VC	97.66 ± 12.81	85.42 ± 14.59	101.00 ± 25.92	96.43 ± 16.40	91.91 ± 14.69	98.16 ± 12.46	94.58 ± 23.56	0.014^a^
TIFF	0.96 ± 0.08	0.99 ± 0.13	0.95 ± 0.10	0.96 ± 0.07	0.97 ± 0.07	0.96 ± 0.06	0.96 ± 0.85	0.770^a^
MEF75%	86.94 ± 18.47	77.59 ± 19.47		85.75 ± 29.65	80.85 ± 17.07		82.15 ± 20.99	0.275^a^
MEF50%	91.00 ± 22.12	78.94 ± 27.28		90.00 ± 29.65	83.06 ± 23.03		84.92 ± 23.92	0.229^a^
MEF25%	88.45 ± 28.34	87.29 ± 32.17		90.06 ± 29.65	88.91 ± 31.82		83.58 ± 37.04	0.950^a^

All results are reported as mean ± standard deviation. M, marathon; HM, half-marathon; FEV1, forced expiratory volume after one second; FVC, forced vital capacity; VC, vital capacity; TIFF, Tiffeneau-Pinelli ratio (FEV1/FVC ratio); MEF25, mean expiratory flow when 25% of VC remain in the lungs; MEF50, mean expiratory flow when 50% of VC remain in the lungs; MEF75, mean expiratory flow when 75% of VC remain in the lungs; baseline, 1–2 days before the run; peak, immediately after the run in the finishing area; recovery, after 2–7 days of recovery.

^a^One-way ANOVA.

**Table 3:** Differences (in liter) between baseline (before the run) and peak (immediately after) lung function testing in 31 marathoners and 33 half-marathoners.

	M		P-value*	HM		P-values*
	EIB+	EIB−		EIB+	EIB−	
FEV1 Diff	−0.67 ± 0.54	−0.17 ± 0.45	0.011	−0.49 ± 0.48	−0.21 ± 0.41	0.135
FVC Diff	−0.88 ± 0.67	−0.20 ± 0.62	0.008	−0.65 ± 0.72	−0.37 ± 0.56	0.274
VC Diff	−0.87 ± 0.89	−0.22 ± 0.82	0.051	−0.06 ± 1.7	−0.14 ± 0.68	0.909

All results are reported as mean ± standard deviation. M, marathon; HM, half-marathon; FEV1, forced expiratory volume after one second; Diff, Difference between baseline and peak values (in liter); FVC, forced vital capacity; VC, vital capacity;

*Unpaired t-test.

#### Forced vital capacity (FVC)

FVC decreased significantly immediately after running a M (FVC [%predicted] M_baseline 100.06 ± 1.99 vs. M_peak 87.61 ± 2.27, p = 0.001) and returned to baseline levels during recovery (FVC [%predicted] M_peak vs. M_recovery 99.19 ± 2.50, p = 0.001) (Table 2). Significant intergroup differences are shown in Fig. 1b.

#### FEV1/FVC ratio

FEV1/FVC ratio was not affected by running M or HM (Table 2). Significant intergroup differences are shown in Fig. 1c.

#### MEF25, MEF50, MEF75

As shown in Table 2 MEF25, MEF50 and MEF75 were unaffected by M or HM.

#### Exercise-induced bronchoconstriction

Performing a spirometry at the finish line we found signs of EIB, in 12 M (35.29%) and 8 HM (22.22%) runners. Among marathoners, we found 8 with mild, 3 with moderate and 1 with severe EIB. 8 half-marathoners suffered from mild bronchoconstriction. Among athletes with EIB, 11/20 (55%) were male and 9/20 (45%) were female. In 16 M and 13 HM runners we could find a decrease of ≥10% in FVC from baseline. In 11 M and 7 HM runners we investigated a decrease in both, FVC and FEV1 ≥ 10% from baseline Table 3 and supplement.

#### Changes in plasma volume

The mean change in plasma volume (%) that occurred in M and HM runners was 1.89 ± 1.88 and −5.38 ± 4.63. Therefore all of the results presented below are corrected for changes in plasma volume.

#### Serum measurements of circulating cytokines in marathoners and half-marathoners

**Figure 2:**
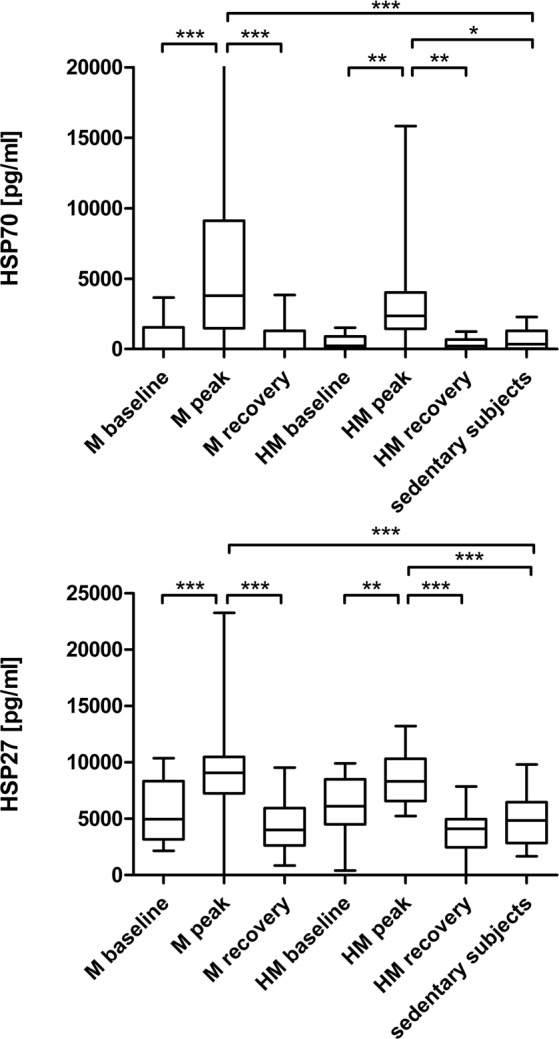
(**a**) Heat shock protein 70 in marathoners and half-marathoners at baseline, peak and recovery compared to sedentary controls. Heat shock protein 70 increased significantly after running a M or HM and returned to baseline concentrations again after 2 to 7 days of recovery. M, marathon; HM, half-marathon; baseline, 1–2 days before the run; peak, immediately after the run in the finishing area; recovery, after 2–7 days of recovery; *p < 0.05, **p < 0.01, ***p < 0.001. (**b**) Heat shock protein 27 in marathoners and half-marathoners at baseline, peak and recovery compared to sedentary controls. Heat shock protein 27 increased significantly after running a M or HM and returned to baseline concentrations again after 2 to 7 days of recovery. M, marathon; HM, half-marathon; baseline, 1–2 days before the run; peak, immediately after the run in the finishing area; recovery, after 2–7 days of recovery; **p < 0.01, ***p < 0.001.

Serum concentrations of HSP family members HSP27 and HSP70: HSP70 increased significantly immediately after running a M and returned to baseline levels during recovery. Baseline serum HSP70 did not differ between M or HM runners and sedentary controls (Table 4). Significant intergroup differences are shown in figure Fig. 2a.

**Table 4:** Serum concentrations of HSP70, HSP27 in 34 marathoners, 36 half-marathoners and 30 sedentary controls before the run (baseline), immediately after (peak) and after 2 to 7 days of recovery (recovery).

	M baseline	M peak	M recovery	HM baseline	HM peak	HM recovery	Sedentary subjects	p-value
HSP27 (pg/ml)	5743.23 ± 2610.80	9470.18 ± 3349.88	4548.75 ± 2418.18	6199.70 ± 2608.28	8467.13 ± 2272.50	4150.57 ± 1986.76	4993.07 ± 2443.95	<0.001^a^
HSP70(pg/ml)	772.75 ± 1223.38	6488.42 ± 6643.95	680.74 ± 1086.03	470.43 ± 473.48	3050.70 ± 2857.98	344.01 ± 378.67	701.87 ± 716.21	<0.001^a^

All results are reported as mean ± standard deviation. M, marathon; HM, half-marathon; baseline, 1–2 days before the run; peak, immediately after the run in the finishing area; recovery, after 2–7 days of recovery; HSP 27, heat shock protein 27; HSP70, heat shock protein 70.

^a^One-way ANOVA.

HSP27 increased significantly in subjects running a M or HM (HSP27 [pg/ml] M_baseline 5743.23 ± 447.75 vs. M_peak 9470.18 ± 592.18, p < 0.001; HM_baseline 6199.70 ± 440.88 vs. HM_peak 8467.13 ± 378.75, p = 0.005) and returned to baseline levels after 2 to 7 days of recovery (HSP27 [pg/ml] M_peak vs. M_recovery 4548.75 ± 434.32, p < 0.001; HM_peak vs. HM_recovery 4150.47 ± 340.73, p < 0.001). Baseline levels were not significantly different in M or HM runners and sedentary controls. Significant intergroup differences are shown in Fig. 2b).

### Correlation of cytokines with running time

Increasing HSP70 serum concentrations in M and HM runners were positively correlated with longer lasting marathon runs (r = 0.308, p = 0.012 and r = 0.295, p = 0.015). HSP 27 did not correlate with running time.

### Correlation of FEV1 with cytokines

Analysing M and HM runners at all time points we found significant correlation of FEV1 with HSP70 (r = −0.209, p = 0.004) and HSP27 (r = −0.160, p = 0.027).

### Serum concentrations of cytokines in EIB

**Figure 3:**
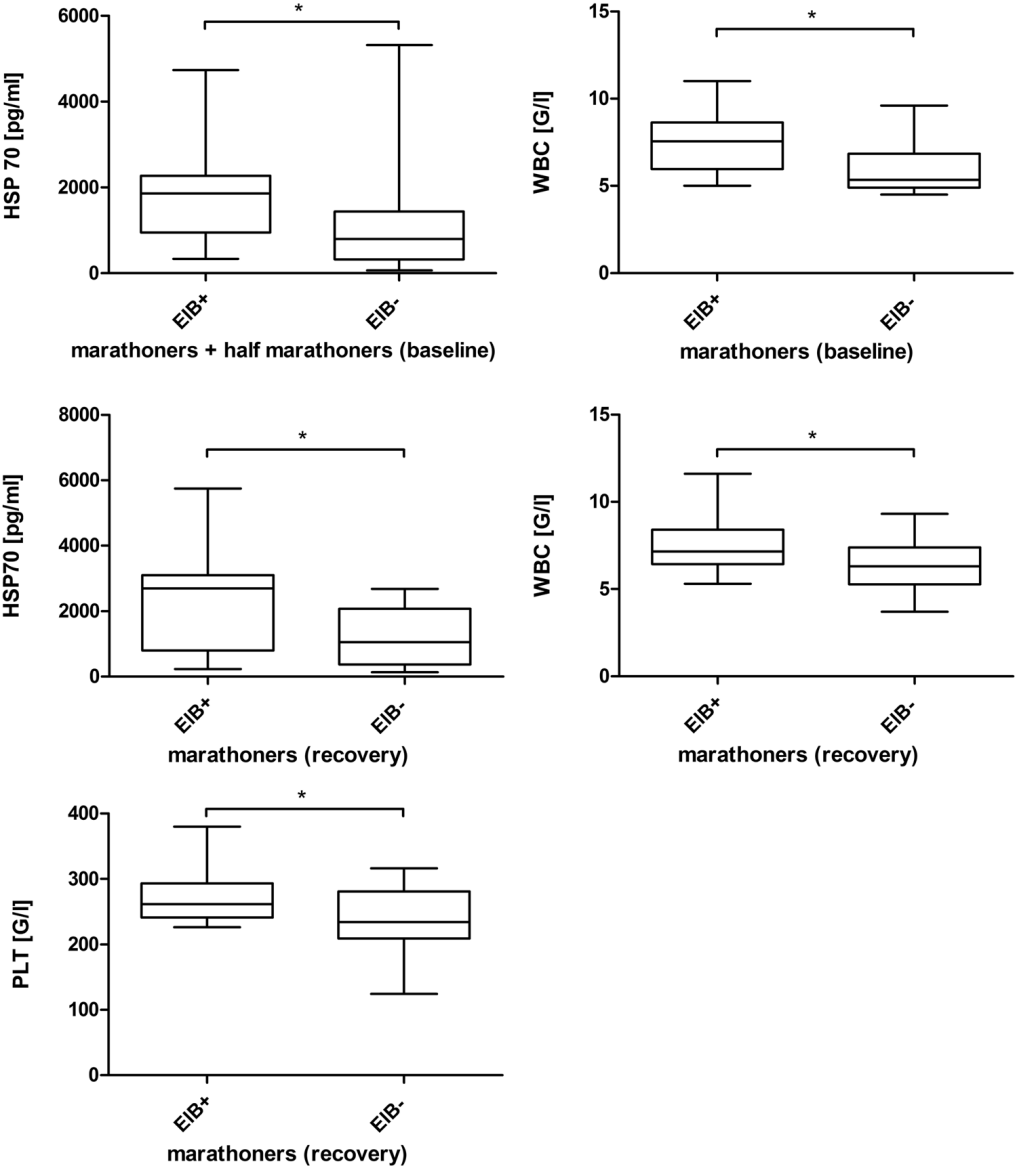
(**a**) HSP70 in marathoners and half-marathoners with and without exercise-induced bronchoconstriction before the competition. HSP70 serum concentrations at baseline are significantly increased in runners showing signs of EIB during a marathon or half marathon compared to runners without EIB. EIB, exercise-induced bronchoconstriction; baseline, 1–2 days before the run; *p < 0.05. (**b**) White blood cell counts in marathoners with and without exercise-induced bronchoconstriction before the competition. White blood cell counts in marathoners at baseline are significantly increased in runners showing signs of EIB during the competition compared to runners without EIB. EIB, exercise-induced bronchoconstriction; baseline, 1–2 days before the run; *p < 0.05. (**c**) HSP70 in marathoners with and without exercise-induced bronchoconstriction after 2 to 7 days of recovery. After 2 to 7 days of recovery, HSP70 serum concentrations in marathoners are significantly increased in runners showing signs of EIB during the competition compared to runners without EIB. EIB, exercise-induced bronchoconstriction; recovery, after 2–7 days of recovery; *p < 0.05. (**d**) White blood cell counts in marathoners with and without exercise-induced bronchoconstriction after 2 to 7 days of recovery. After 2 to 7 days of recovery, white blood cell counts in marathoners are significantly increased in runners showing signs of EIB during the competition compared to runners without EIB. EIB, exercise-induced bronchoconstriction; recovery, after 2–7 days of recovery; *p < 0.05. (**e**) Platelets in marathoners with and without exercise-induced bronchoconstriction after 2 to 7 days of recovery. After 2 to 7 days of recovery, platelets in marathoners are significantly increased in runners showing signs of EIB during the competition compared to runners without EIB. EIB, exercise-induced bronchoconstriction; recovery, after 2–7 days of recovery; *p < 0.05.

After distributing runners in a group with EIB and without EIB, we found significantly higher HSP70 serum concentrations in runners with EIB on the day before the competition (Table 5, Fig. 3a). After analysing marathoners and half marathoners separately, we found significantly increased WBC in marathoners showing signs of EIB before participating in the competition (Fig. 3b). Immediately after the race we could not detect any cytokine changes between runners with and without EIB. After 2–7 days of recovery significantly increased HSP70 serum concentrations (Fig. 3c), WBC (Fig. 3d) and PLT (Fig. 3e) were found in marathoners with EIB, when compared to marathoners without EIB.

**Table 5:** Serum measurements in exercise-induced bronchoconstriction in marathon and half marathon runners.

	M baseline			M recovery		
	EIB+	EIB−	p-value	EIB+	EIB−	p-value
HSP70	987.47 ± 1486.70	655.63 ± 1073.97	0.143^c^	2539.27 ± 1692.52	1237.24 ± 835.27	0.032^c^
WBC	7.41 ± 1.74	6.02 ± 1.51	0.021^c^	7.69 ± 1.87	6.44 ± 1.61	0.048^c^
PLT	282.50 ± 73.26	248.05 ± 53.35	0.383^c^	273.00 ± 43.06	237.27 ± 48.39	0.040^c^
	**M + HM baseline EIB +**			**M + HM baseline EIB −**		**p-value**
HSP70	1856.52 ± 1225.64			1106.99 ± 1074.34		0.014^c^

All results are reported as mean ± standard deviation.

M, marathon; HM, half.-marathon; baseline, 1–2 days before the run; recovery, after 2–7 days of recovery HSP70, heat shock protein 70**;** WBC, white blood cells; PLT, platelets; +EIB, athletes with exercised induced bronchoconstriction; −EIB, athletes without exercise induced bronchoconstriction.

^c^Independent-samples t-test.

The molecules HMGB1, sRAGE, esRAGE, AGE-CML, ST2, IL33 and IL1-RA remained unchanged between athletes with EIB compared to runners without EIB.

### Correlation of FEV1 with cytokines in EIB

Increasing HSP70 serum concentrations in M and HM runners were positively correlated with FEV1 immediately after the race (r = −0.476, p = 0.034).

## Discussion

This is the first study investigating HSP serum concentrations in non-professional non-asthmatic marathon and half marathon runners at three different time points and correlating it with lung function. In this field test, signs of EIB in M and HM runners were found in 12/34 (35.29%) and 8/36 (22.22%), respectively. In elite athletes a prevalence of asthma and exercise-induced bronchoconstriction between 30% and 70% was reported^10^. In few studies it appears that EIB does not impact athletic performance^28^. Although prevalence of undiagnosed EIB seems high in athletes^29^ and a number of organizations recommend screening in the general population^30^ and athletes^31,32^, the evidence about benefits, harms, and burdens of screening for EIB is insufficient.

The current study not only observed significant declines in FEV1 finishing the M or HM but also significant reduction in measured FVC. The field study design does not meet the recommended diagnostic algorithm for the physician performing the diagnosis in the primary care setting. Athletes reporting symptoms suggestive of EIB should undergo spirometry and physical examination. In athletes diagnosed with obstruction on spirometry a bronchial provocation test followed by reversibility testing with a pharmacological bronchodilator will support the diagnosis of EIB^33^. For details refer to the ATS/ERS statement^28^. The observation of falls in FVC directs the attention to phenomena other than EIB that cause exercise-induced dyspnoea. The possibilities range from athletes reaching their physiological limits (deconditioned or unfit athletes), organic pathologies other than EIB, functional or structural forms of dysfunctional breathing and other pulmonary and non-pulmonary pathologies (arrhythmia, intracardiac shunts)^34,35^. The field test may be particularly prone to blur the picture of EIB in athletes who may have even sprinted to the finish line and also present with fatigue. The spirometry in our setting was done between 5 and 15 minutes after reaching the finish line to allow some recovery of the athletes in order to discover EIB and not only fatigue.

There was no significant correlation between decreased FEV1 and training frequency or training extent in our study cohort. Investigating the impact of urban living it could be demonstrated that a fall in FEV1 after exercise of ≥10% occurred in 22.9% of urban children and 13.2% of rural children^8^. Exposure to pollution, such as freshly generated emissions with particulate matter <100 nm and ozone is suspected most harmful^36^. Although 78.94% of our participants experiencing EIB live in cities with more than 25000 inhabitants which could be a possible explanation for EIB development. The training environment (air pollution) may have been a factor in EIB development but we did not measure this directly. For athletes living in the urban environment, training during low traffic hours and in low traffic areas is recommanded^36^.

The pathogenesis of EIB is still poorly understood. Several risk factors for the development of EIB in non-asthmatic athletes have been reported, such as environmental conditions^12^ and type of exercise^37^.

Endurance athletes, especially winter sport athletes and swimmers have a higher probability of developing EIB than other athletes and the general population^36^. One possible explanation may be that mouth breathing allows an increased penetration of ozone, particulate matter, and other pollutants^36^.

Behavioural modifications such as completing interval or a combination warm-up exercise before planned exercise, avoidance of cold weather or use of devices, dietary modifications with low salt-diet, supplementary fish oil, lycopene or ascorbic acid are recommended^29^. Interestingly, EIB and asthma seem to be more common in distance runners than in the general population, associated with a higher exposition to traffic^38,39^. As baseline therapy for EIB inhaled corticosteroids are recommended, which is considered the most effective anti-inflammatory therapy^40^. Short acting β2-agonists can be added as prophylaxis or during acute bronchoconstriction. These agents stimulate β2-receptors on airway smooth muscle leading to muscle relaxation and bronchodilation^41^. There is some evidence that β2-agonists prevent mast cell degranulation^41^. This medication is inhaled 5–20 minutes before exercise and usually protects against or attenuates EIB for two to four hours^42,43^. These agents may fail to prevent bronchoconstriction in 15–20% of patients with asthma^44^. Long acting β2 agonists are often used for severe cases^29^. The World Anti-Doping Agency (WADA) has prohibited all β2 agonists in and out the competition. Exemptions from this are formoterol, salbutamol, salmeterol and terbutaline^45^.

A role of HSPs in sports has already been described in treadmill running leading to increased serum HSP72 concentrations^20^, in strength training elevating HSP27 and HSP70 in musculi vastus lateralis and trapezius^46^, in half marathoners with increased HSP transcripts and their corresponding proteins^47^, in half marathoners with a greater percentage of HSP27, HSP60 and HSP70 expression in leucocytes^48^ and in ultra-marathoners with increased HSP70 and HSP32 mRNA expression^49^. Both, the amount of HSP 27 and 70 expressing leucocytes and the fluorescence intensity of both HSPs in monocytes increased significantly after running a HM. Simultaneously, a down regulation of HSP-positive cells could be demonstrated in 12 trained athletes at rest when compared to 12 untrained persons at rest^48^. In our study cohort we did not detect different serum concentrations of HSP 27 and 70 comparing M and HM runners, and sedentary controls. There was neither a difference at baseline nor during the recovery period contrasting the above described cellular adaptive changes. HSP 27 and 70 mRNA concentrations increased similarly after running a HM^47^. These data are in line with significantly increased serum HSP27 and 70 concentrations immediately (at the finish line) after running a M of HM.

This is the first study investigating HSP in EIB in recreational marathon and half marathon runners. HSP70 has been associated with an increased risk of COPD in coal workers and has been identified as a potential monitoring marker for COPD in coal workers^50^. When investigating patients with persistent bronchial asthma plasma HSP70 has been found to serve as useful markers to estimate the degree of airway obstruction in these patients^51^. Further, pregnant patients with asthma had significantly increased HSP70 concentrations when compared to healthy pregnant women^52^. We found significantly increased HSP70 serum concentrations in all runners with EIB compared to those without EIB. There are several possible hypotheses for the role of HSP70 in EIB. First, HSP70 is a known member of the molecular chaperone family, which is involved in protecting cells against physical or chemical stress^53^. Increased serum HSP70 concentrations indicate systemic inflammation and oxidative stress^54^. Another explanation for the involvement of HSP70 in the pathogenesis of asthma may be HSP70 mediated decreases in the production of interleukin 1 and tumor necrosis factor α^55,56^. In patients with asthma HSP 70 has been reported to be involved in antigen processing^57^.

A skin temperature above 35 °C is associated with greater cardiovascular strain for a given core temperature and therefore results in earlier exhaustion. If skin is relatively cool, higher core temperatures can be better sustained during exercise. It is still unknown whether this effect results from extensive heat acclimatization, training practices, natural selection or a combination of them^58^. Skin temperatures after the race were significantly lower at all sites in M and HM runners after the race, than at rest. A fall in skin temperature during exercise has been described by several investigators, whereby the extent of temperature drop correlates with the level of intensity during exercise and the individual fitness^59,60^. Furthermore, temperature loss correlates positively with thinner skin-fat folds. We found significantly lower skin temperatures at all sites in M and HM runners at rest, when compared to sedentary controls. A possible explanation could be different body composition and the different thermal properties of tissues underlying the skin of runners^59^.

Interestingly, mechanisms of RAGE axis molecules described in asthma are not involved in EIB in non-asthmatic marathon and half marathon runners.

## Conclusion

We found an unexpectedly high proportion of EIB immediately after the event. In symptomatic non-professional athletes EIB is a likely diagnosis that should not be overseen in order to allow sufficient information and possible therapy and avoidance of exercise and sports events that may contribute to overall health. HSP70 serum concentrations and elevated WBC could indicate a predisposition to EIB. Further studies of the role of HSPs in the pathophysiology of EIB are warranted.

## Material and Methods

### Setting

In this study male and female volunteers running a full or half marathon (Vienna City Marathon, held on April 15th, 2012) were included and assessed the day before the competition (at the time of registration, further referred to as baseline), immediately after the marathon at the finish area (peak) and two to seven days after (recovery). A spirometry was performed by a portable lung function testing device (PC Spirometry, SDS 104, Schiller AG, Linz, Austria) to assess FVC%, FEV1% and the FEV1/FVC ratio at all three time point. On this day mean air pressure was 974.8 hPA, mean air temperature was 10.8 °C, mean relative air humidity 87.3, wind direction was northwest, wind force was 12–19 km/h and there was no precipitation^61^. Mean ozone half-hourly means between 9am and 3 pm was 48.04 µg/m³ ^62^.

Patients were studied for exercise-induced bronchoconstriction (EIB), defined as a decrease of ≥10% in FEV1 from baseline and can be graded as mild (FEV1 decrease ≥10% but <25%), moderate (≥25% but <50%) and severe (≥50%)^29^.

None of the study participants suffered from asthma, COPD, or reported respiratory symptoms related to exercise. None of the participants presented with COPD as defined by the global initiative for chronic obstructive lung disease (GOLD) criteria^63^. Bronchodilators were neither administered for study purposes nor taken by study subjects as assessed by our standardized study questionnaire.

The number of Smoker:Nonsmoker:Neversmoker was 01:08:25 for the M runners, 02:06:28 for HM runners and 03:04:23 for the controls. The number of pack years (PY) was 1.70 ± 0.85 PY in marathoners, 3.23 ± 1.28 half-marathoners and 3.32 ± 1.86 in the control group.

Ethical approval was obtained from the institutional review board (EK 1034/2012) of the Medical Universty of Vienna. All tests were performed in accordance with the Declaration of Helsinki and the guidelines for good scientific practice of the Medical University Vienna. All subjects participating in this study gave written informed consent.

### Laboratory procedures

Blood samples were collected at baseline, peak and recovery. Blood was taken from the antecubital vein in EDTA and serum gel tubes. EDTA tubes were used for blood count analysis using a haematology analyser (Sysmex KX-21N) and for investigations of routine parameters. Serum was separated by centrifugation (15 minutes at RCF 2845 × g) and used for quantification of the indicated proteins. All samples were stored at −80 °C within 2 hours after the event.

The study was conducted at the research laboratories oft he department of thoracic surgery at the Medical University Vienna.

### Temperature measurements

Core temperature was measured at baseline and peak by sliding a temporal scanner (Exergen, Gepa-Med Medizintechnik GmbH, Austria) thermometer straight across the forehead. Skin temperature was assessed at the abdomen, the upper arm, the upper leg and lower leg using a DermaTemp Infrared Surface Skin Scanner (Exergen, Gepa-Med Medizintechnik GmbH, Austria).

### Detection of proteins in serum and urine

Commercially available enzyme-linked immunosorbent assays (ELISA) were used to measure serum and urine concentrations of the indicated proteins. These trials were performed according to the manufacturers’ instructions. The employed quantitative immunoassays were purchased as follows: HSP70 (RnD Systems, Minneapolis, MN, USA), HSP27 (RnD Systems, Minneapolis, MN, USA), sRAGE (RnD Systems, USA), esRAGE (B-Bridge International Inc., USA), HMGB1 (IBL International GmbH, Germany), Advanced glycation endproducts carboxymethyllysine (AGE-CML) (MicroCoat Biotechnologie GmbH, Germany), ST2 (RnD Systems), IL1-RA (RnD Systems) and IL33 (RnD Systems).

Researchers performing the laboratory work and data analyses were blinded to the study groups associated with each sample.

### Correction for changes in plasma volume

All measurements were corrected for changes in plasma volume due to dehydration as already described before^26^.

### Statistical methods

Statistical analysis of data was performed using SPSS software (version 24; IBM SPSS Inc., IL, USA) and the SAS System (version 9.4; SAS Institute Inc., Cary, NC, USA). To compare means of two or more than two independent groups with normal distributions unpaired student’s t tests and one-way ANOVA were used, respectively. Non-normal distributions were evaluated using Kruskal-Wallis rank test. The type of used test is indicated in the table and/or the results section. Post hoc comparisons were computed with Tukey’s-B and Bonferroni correction. All data are reported as mean ± standard deviation in the text and as mean (median) ± standard deviation (standard error of the mean) in tables. To analyse frequencies or distributions of nominal values in two or more groups chi-square test for independence was applied.

For evaluation of linear relationships between two numerical measurements made on the same subjects (e.g.: correlation between HSP serum concentrations and the M time) pearson product moment was used. Results are as Pearson product-moment correlation coefficient, also known as r. Figures were created using GraphPad Prism 6 (GraphPad Software Inc., California, USA).
