# Publisher Correction: Exercise-induced bronchoconstriction, temperature regulation and the role of heat shock proteins in non-asthmatic recreational marathon and half-marathon runners

**DOI:** 10.1038/s41598-019-48897-5

**Published:** 2019-08-22

**Authors:** Christine Bekos, Matthias Zimmermann, Lukas Unger, Stefan Janik, Andreas Mitterbauer, Michael Koller, Robert Fritz, Christian Gäbler, Jessica Didcock, Jonathan Kliman, Walter Klepetko, Hendrik Jan Ankersmit, Bernhard Moser

**Affiliations:** 1 Christian Doppler Laboratory for Cardiac and Thoracic Diagnosis and Regeneration, Vienna, Austria; 2 Medical University of Vienna, Comprehensive Cancer Center, Department of Obstetrics and Gynecology, Division of General Gynecology and Gynecologic Oncology, Vienna, Austria; 3 Sportordination, Alserstraße 28, Vienna, Austria; 4 Department of Surgery, Division of Thoracic Surgery, Medical University Vienna, Vienna, Austria

Correction to: *Scientific Reports* 10.1038/s41598-019-39983-9, published online 12 March 2019

In the original version of this Article, the author Hendrik Jan Ankersmit was incorrectly indexed. This error has now been corrected.

